# Recurrent Vulvovaginal Candidiasis: Could It Be Related to Cell-Mediated Immunity Defect in Response to *Candida* Antigen?

**DOI:** 10.22074/ijfs.2017.4883

**Published:** 2017-09-03

**Authors:** Zahra Talaei, Saba Sheikhbahaei, Vajihe Ostadi, Mazdak Ganjalikhani Hakemi, Mohsen Meidani, Elham Naghshineh, Majid Yaran, Alireza Emami Naeini, Roya Sherkat

**Affiliations:** 1 Acquired Immunodeficiency Research Center, Isfahan University of Medical Sciences, Isfahan, Iran; 2 Cellular and Molecular Immunology Research Center, Isfahan University of Medical Sciences, Isfahan, Iran; 3 Infectious Diseases and Tropical Medicine Research Center, Isfahan University of Medical Sciences, Isfahan, Iran; 4 Department of Obstetrics Gynecology, Isfahan University of Medical Sciences, Isfahan, Iran

**Keywords:** Allergy, *candida albicans*, Cell Mediated Immunity, Vulvovaginal Candidiasis, Atopy

## Abstract

**Background:**

Recurrent vulvovaginal candidiasis (RVVC) is a common cause of morbidity affecting millions of women worldwide. Patients with RVVC are thought to have an underlying immunologic defect. This study has been established to evaluate cell-mediated immunity defect in response to *candida* antigen in RVVC cases.

**Materials and Methods:**

Our cross-sectional study was performed in 3 groups of RVVC patients (cases), healthy individuals (control I) and known cases of chronic mucocutaneous candidiasis (CMC) (control II). Patients who met the inclusion criteria of RVVC were selected consecutively and were allocated in the case group. Peripheral blood mononuclear cells were isolated and labeled with CFSE and proliferation rate was measured in exposure to candida antigen via flow cytometry.

**Results:**

T lymphocyte proliferation in response to *candida* was significantly lower in RVVC cases (n=24) and CMC patients (n=7) compared to healthy individuals (n=20, P<0.001), but no statistically significant difference was seen between cases and control II group (P>0.05). Family history of primary immunodeficiency diseases (PID) differed significantly among groups (P=0.01), RVVC patients has family history of PID more than control I (29.2 vs. 0%, P=0.008) but not statistically different from CMC patients (29.2 vs. 42.9%, P>0.05). Prevalence of atopy was greater in RVVC cases compared to healthy individuals (41.3 vs. 15%, P=0.054). Lymphoproliferative activity and vaginal symptoms were significantly different among RVVC cases with and without allergy (P=0.01, P=0.02).

**Conclusion:**

Our findings revealed that T cells do not actively proliferate in response to Candida antigen in some RVVC cases. So it is concluded that patients with cell-mediated immunity defect are more susceptible to recurrent fungal infections of vulva and vagina. Nonetheless, some other cases of RVVC showed normal function of T cells. Further evaluations showed that these patients suffer from atopy. It is hypothesized that higher frequency of VVC in patients with history of atopy might be due to allergic response in mucocutaneous membranes rather than a functional impairment in immune system components.

## Introduction

Vulvovaginal candidiasis (VVC) is the second most common cause of genital tract infections (1). It has been shown that VVC affects 75% of female population at least once during their lives and 5-10% at higher frequencies (2). In more than 85% of the cases, VVC is primarily caused by *candida* albicans, followed by *candida glabrata* with an incidence of 4-5%, and to a lesser extent by *candida tropicalis* and *candida parapsilosis *(3, 4). Recurrent VVC (RVVC) is defined as occurrence of four or more episodes of VVC during 12 months (5). Several risk factors have been proposed for RVVC including pregnancy, diabetes mellitus, corticosteroid therapy, antibiotic therapy, and some hygiene habits. Primary impaired immune response can also be considered as a predisposing factor in RVVC patients with none of the risk factors above (6-9). Our knowledge about immune response against fungal pathogens has advanced considerably in recent years. A low rate of immune cell proliferation following antigenic or mitogenic stimulation is assumed to be an indicator for immunodeficiency diseases in clinical and research projects (10). Previous studies showed reduction in *candida*-related lymphocyte proliferation in RVVC patients, focusing on cellular immunity involvement in the process of VVC (11-13). Anergy to *candida* in in vivo skin test of RVVC patients is in accordance with impaired T cell proliferation in these patients (14). However, paradoxical evidences indicate no difference in lymphocyte transformation, leukocyte migration inhibition and lymphokines produced by Th1 cells among RVVC patients and healthy individuals (15, 16).

There are several ways to evaluate cellular immunity against *candida* infection. Among the different laboratory methods to test T lymphocyte proliferation stimulated by *candida* antigens flow cytometry using carboxy fluorescein diacetate succinimidyl ester (CFSE), is an established method describes cell division with simple intuitive and meaningful parameters. CFSE provides clear tracking of cell division via a 50% concentration reduction of the fluorescent dye in each divided cell. In the case of T cells this fluorescence reduction may be detected after 5 days. The goal of the current study was to measure T cell proliferation stimulated by *candida* antigen in RVVC patients in comparison with healthy controls and chronic mucocutaneous candidiasis (CMC) subjects using CFSE. Moreover, our aim was to introduce a simple and cost effective diagnostic test to be used routinely in patients suffering from RVVC.

## Materials and Methods

Our cross-sectional study was performed from January 2014 till May 2015. Patients with 4 or more episodes of VVC infection during the past year who were initially visited by gynecologists and referred to immunology clinics were enrolled in RVVC case group. All episodes of RVVC were confirmed with vaginal swab smear and culture. Control I subjects were healthy individuals without history of vulvovaginitis during the past year and also had less than 3 episodes of vulvovaginitis in a year during the previous years. Age and educational level did not vary among cases and individuals in control I group. Patients with chronic, persistent or recurrent non-invasive mucocutaneous candidiasis associated with organ infections, autoimmunity, vasculopathy and absence of predisposing conditions such as diabetes or HIV were enrolled in the study as control II group (CMC patients) (17).

Patients with pregnancy, history of using any antibiotic, corticosteroid, hormone therapy, antifungal within the past 30 days and medical history of diabetes mellitus were excluded. Also patients with refractory VVC were excluded because RVVC means episodes of candida infection, with complete response to treatment each time. Required information including age, education status, family history of primary immunodeficiency diseases (PID) (in 1st, 2^nd^ or 3^rd^ degree relatives), history of allergy (confirmed by the clinical immunology and allergy specialist), history of hypothyroidism, history of using antifungal and frequency of VVC within last year was collected using a questionnaire. PID is defined as a heterogeneous group of diseases with higher susceptibility to infections as a result of immunity defect. International Union of Immunological Societies classifies PIDs in 8 large categories according to the impaired components of immune system (18). Severity of vaginitis was measured with a semiquantitative basis scoring from 0-3: 0 (absent), 1 (mild), 2 (moderate), 3 (severe). Sign and symptoms like pruritus, erythema, burning, edema and excoriation/fissure have been scored according to the patient’s statement. The sum-score of <4 is considered as asymptomatic/mild vulvovaginitis and excluded from our study and total score of >7 is defined as severe vulvovaginitis (19). Written and signed informed consents were obtained from all participants. The study was approved by Ethical Committee of Isfahan University of Medical Sciences (reference number: 283457). All enrolled patients were assisted by only one clinician.

At first, prior to blood sampling, phytohemagglutinin (PHA)-induced skin test was done in patients and control II group as an index of cell-mediated immunocompetence. By this test the mitogen PHA is injected subcutaneously and the swelling is measured 24 hours later. Blood samples were taken from the subjects. White blood cell count, immunoglobulin level and basic immunological markers were measured. Peripheral blood mononuclear cells (PBMC) were isolated using Ficoll-hypaque gradient separation (Amersham Biosciences, Germany). The number of PBMCs was set at 5-10×106 million cells per milliliter in PBS (Cayman Kit, Canada), were labeled with CFSE (Cayman Kit, Canada), and incubated for 30 minutes at 37°C with 5% CO_2_. Cells were then centrifuged and the supernatant was discarded. Cell pellet was re-suspended in RPMI-1640 culture medium containing 10% fetal calf serum (FCS) and incubated again at 37°C with 5% CO_2_ (Cyman kit, Canada). Triplicate cultures of 2×10^5^ cells in 200 μl medium per well were established in 96-well round-bottomed cell culture plates. The candida antigen (HollisterStier, Germany) was diluted at a ratio of 1 to 10 V/V in RPMI-1640 culture medium containing 10% FBS and added to the wells containing the cell suspension. After addition of the antigen, cell plate was incubated at 37°C and 5% CO_2_. After 5 days, the cells were transferred to microtubes and washed with PBS. Finally, Proliferation was evaluated with flow cytometery (Partec, Denmark) using the FloMax software. The assays were done in totally blinded manner. Statistical analyses were done by Student’s t test, ANOVA and chi square test using SPSS16 software program (SPSS Inc., Chicago, IL, USA).

## Results

**Fig.1:**
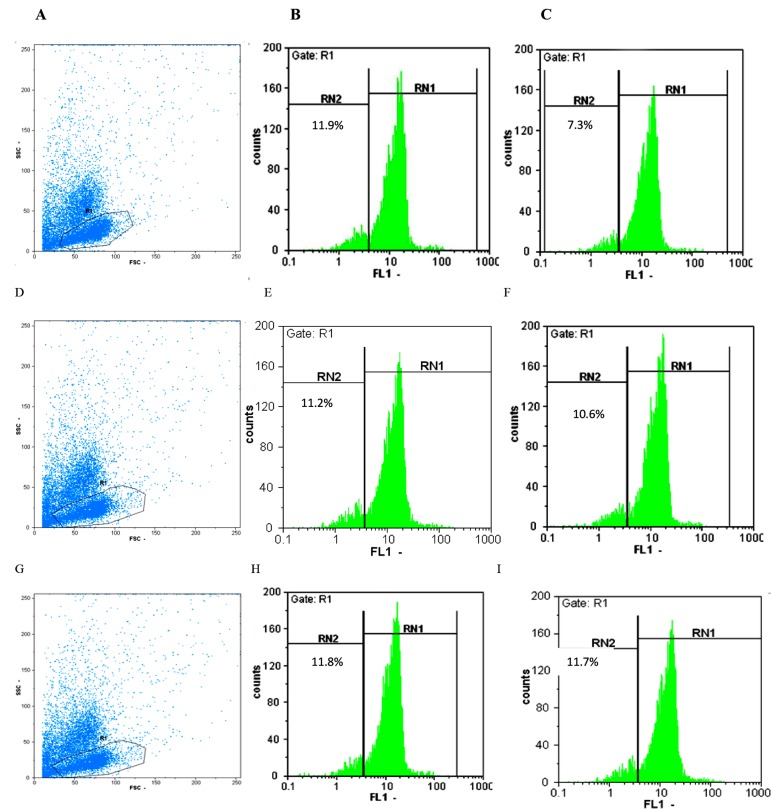
Plots of flow cytometry in recurrent vulvovaginal candidiasis (RVVC), healthy individuals (control I) and chronic mucocutaneous candidiasis (CMC) patients (control II). **A.** CFSE- labeled lymphocytes in a healthy control after 5 days, **B.** Proliferation of lymphocytes after 5 days with antigen in a healthy control, **C.** Proliferation of lymphocytes after 5 days without antigen in a healthy control, **D.** CFSE-labeled lymphocytes in a RVVC case, **E.** Proliferation of lymphocytes after 5 days with antigen in a RVVC case, **F.** Proliferation of lymphocytes after 5 days without antigen in a RVVC case, **G.** CFSE- labeled lymphocytes in a CMC case, **H.** Proliferation of lymphocytes after 5 days with antigen in a CMC case, and **I.** Proliferation of lymphocytes after 5 days without antigen in a CMC case.

Twenty-eight patients with RVVC, 28 healthy individuals (control I) and 7 patients with chronic mucocutaneous candidiasis (control II) entered the study. Four RVVC cases and 8 healthy subjects were excluded; hence 24 cases, 20 controls and 7 CMC cases enrolled the study. Characteristics of individuals in each group are shown in Table 1. Age and educational level were not different among the 3 groups. Immunoglobulin level, white blood cell (WBC) counts, immunological biomarkers and PHA skin test were all normal among controls and patients. Mean proliferation of T lymphocytes in response to *candida* antigen was 1.89 ± 1.6 in cases, 3.94 ± 1.0 in healthy controls and 0.81 ± 0.42 in CMC patients (Fig .1).

**Table 1:** Characteristic of individuals divided in 3 groups of recurrent vulvovaginal candidiasis (RVVC) cases, control and chronic mucocutaneous candidiasis (CMC)


		RVVC casen=24	Control I n=20	Control II (CMC) n=7	P value

Age (Y)		33.3 ± 8.6	32.8 ± 7.9	29.1 ± 8.4	0.5
Educational status					0.3
	Lower than high school	15 (62.5%)	8 (40%)	4 (57.1)	
	Higher than high school	9 (37.5%)	12 (60%)	3 (42.9%)	
Family history of PID					0.013
	Yes	7 (29.2%)	0	3 (42.9%)	0.008 (post hoc case-control I )
	No	17 (70.8%)	20 (100%)	4 (57.1%)	0.49 (post hoc case-control II)
History of atopy					NS
	Yes	10 (41.7%)	3 (15%)	1(14.3%)	0.054 (post hoc case-control II)
	No	15 (58.3%)	17 (85%)	6 (86.6%)	
Drug history (antifungal)					
	Yes	15 (60%)	0	2 (28.6%)
	No	10 (40%)	20 (100%)	5 (71.4%)
Clinical symptom severity (mean ± SD)		5.8 ± 1.5	-	6.8 ± 1.3	0.1


PID; Primary immunodeficiency diseases.

Although T cell response was significantly different among the 3 groups (P<0.001), it did not differ statistically between cases and CMC patients (P>0.05). Family history of PID was seen in 29.2% of cases, 42.9% of CMC patients and none of the healthy individuals. History of PID in family members was significantly different among the groups (P=0.01). The prevalence of allergy in RVVC cases was higher than control II group (P=0.054, 41.3 vs. 15%). The median of recurrence in patients during last year was 5.5 times with 4 times as minimum and 8 times as maximum episodes of recurrences. Vaginal symptoms in RVVC cases were not different from CMC group (P>0.05). T cell proliferation was negatively correlated with frequency of RVVC and clinical symptom severity respectively (r=-0.7, P<0.001, r=-0.4, P=0.013). T cell activation was greater in RVVC cases who had allergy compared to the ones without allergy (P=0.01) and also in patients who had used antifungals in comparison with patients who did not have the history of using antifungals (P=0.057). Vaginal clinical symptoms were different in cases with or without allergy and cases with or without history of using antifungal agents (Table 2).

**Table 2:** T cell proliferation and vaginal symptom severity in patients with/without atopy and in patients with/without history of antifungal consumption


		n	T cell proliferation (mean ± SD)	P value	Clinical symptom severity (mean ± SD)	P value

Atopy in RVVC cases				0.01		0.02
	Yes	10	2.90 ± 1.5		5.0 ± 1.1	
	No	14	1.27 ± 1.31		6.4 ± 1.5	
Antifungal				0.57		0.005
	Yes	16	1.53 ± 0.89		5.3 ± 1.2	
	No	15	0.93 ± 0.78		6.8 ± 1.5	


RVVC; Recurrent vulvovaginal candidiasis.

## Discussion

VVC is a fungal infection predominantly caused by *candida albicans*. Despite large number of surveys on mechanisms involving localized vaginal yeast infections, the pathophysiology is not determined yet. Clinical studies demonstrated the role of both innate and adaptive immunity in VVC (20). Both arms of adaptive immunity (cell mediated immunity and humoral immunity) are thought to be protective against *candida* infection (21). A study found infiltration of T cells predominantly in vaginal fluid of fungal infected rats; however, another study showed proliferation of vaginal B-lymphocytes isolated from *candida*-infected rats in response to fungal antigen (22).

There is considerable conflict about susceptibility to RVVC in the literature, whether it is mainly due to impairment in T cell function. A study done by Corrigan et al. (23) revealed that subjects with RVVC have decreased T cell proliferation and IFN-γ secretion in stimulation with *candida*. Alternative clinical studies detected significant decrease in lymphoproliferative activity of helper T cells and pro-inflammatory cytokines (24, 25). It is necessary to notice that response to subcutaneous injection of PHA in RVVC patients and healthy individuals did not differ. The test is an approval document indicating that T cells are responsive and have normal function in exposure to other antigens. Our results showed that T cells did not proliferate normally in 58% of RVVC cases compared to control I group. However there were RVVC cases (42%) who had normal LTT but were suffering from allergy at the same time or had the history of allergic reactions. So RVVC patients with allergies showed higher T cell proliferation than RVVC patients without allergies. Our findings suggest two hypotheses for host defense against recurrent *candida* infection. One emphasizes on the importance of T cell mediated defect in response to *candida* predisposing vaginal tissue to yeast infection, and the other one proposes an underlying immune hypersensitivity reaction in vaginal mucosa rendering the signs and symptoms of vulvovaginitis in allergic patients who had normal T cell function. We indicated that sever form of VVC is related to lower proliferation of T cells, as higher clinical score was seen in patients with cell-mediated immunity (CMI) defects in response to *candida* than patients with normal T cells.

Early clinical studies have shown that defects in CMI by Th1 cells lead to recurrent fungal infections (26-28). It has been revealed that mutations affecting Th17/IL17 increase susceptibility to CMC and was confirmed with results of the study assessing vaginal yeast problems in response to inhibition of Th17 (29). These results are consistent with our findings while they are in contrast with some other studies, which reported normal cellular immunity in evaluation of RVVC patients (15, 30, 31). Controversial results have been published about contribution of Th2 cells in protection against *candida* infection. Some studies propose no immunologic role of Th2 and secretory cytokines (32, 33), while other studies report higher levels of Th2-related chemokines in vaginal fluid (34, 35).

Some of the studies conducted on the relationship between patient’s immunity and the incidence of VVC have focused on the evaluation of local safety and allergic reactions in the vaginal environment. Several evidences exist suggesting that RVVC has strong correlation with atopy (36- 39). Treatment with zafirlukast, cetirizine or other allergy immunotherapy medicines induce remission and are sometimes considered as maintenance therapy in patients who failed to get resolution of symptoms by variant antifungal treatments (40, 41). Weissenbacher et. al. (42) have studied immunological factors including IL-4, IL-5, IL- 13 and PGE2 in vaginal discharge in women with RVVC proposing that infected cases had a specific local immune deficiency in that area. Another study evaluating patients with hypersensitivity to their spouse’s seminal plasma proteins suggests that IgE-mediated immune responses may be involved in this process (43). We concluded that the etiology of RVVC in allergic patients is an inflammatory response to allergens in different mucosal membranes (oropharynx, sinus and vagina), which provide vaginal environment susceptible to fungal growth.

Other studies have demonstrated that maintenance therapy with antimycotic drugs is effective in lowering sign and symptom severity and frequency of recurrence (44, 45). We found that patients with history of using antifungal agents did not have significantly higher lymphoproliferative activity but presented milder symptoms than the groups not having received such drugs. In our studies patients taking antifungal medicine in the past 30 days were excluded and only patients who had the history of receiving antimycotics prior to that were analyzed. Previous *in vitro* studies performed by Fidel et al. (46, 47) found that immunity against *candida* is not mediated by systemic host defense and it is mostly associated with local acquired mucosal immunity.

As opposed to this publication, a study done by Leigh et al. (48) indicated that the incidence of mucosal infection was not different between HIV+ patients and healthy persons. However, another study evaluating HIV+ patients with oral and vaginal candidiasis, suggested that immunity to *candida* is mediated by immunity from both systemic and local sources (49). Further studies demonstrated that reduced protection against *candida* is mostly due to *candida albicans*-specific adaptive immunity (23, 50). As we extracted lymphocytes from peripheral blood, it would be an evidence of systemic immunity involvement in this process. Our study did not assess local immunity to *candida* so it could not reveal the role of local immunity in developing RVVC. Other hypotheses about susceptibility to candida infection are increased polymononuclear leukocyte (PMN) response and chemotactic factors (23, 51), impaired innate immune response (52) and defect in Th17 response to *candida* due to dectin-1 mutation (53).

## Conclusion

The result of this study showed that the patients with RVVC could have had a pre-existing defect in their CMI or a proven history of allergy, which could increase the susceptibility of mucosa to get *candida* infection. Since T cell mediated immunity seems to have an important role in development of RVVC, diagnosis and treatment of this infection should be performed with regard to T cell immunity. LTT is recommended to be employed routinely using CFSE staining and measured by flow cytometry in patients who complain about recurrent incidents of *candida* vulvovaginitis. By further studies confirming our results, RVVC may be classified as CMC, one of the PIDs, which should then receive treatment required for CMC patients, while others with simultaneous history of allergy could take benefit from allergy treatments.
